# Towards reconstruction of the lost Late Bronze Age intra-caldera island of Santorini, Greece

**DOI:** 10.1038/s41598-018-25301-2

**Published:** 2018-05-04

**Authors:** Dávid Karátson, Ralf Gertisser, Tamás Telbisz, Viktor Vereb, Xavier Quidelleur, Timothy Druitt, Paraskevi Nomikou, Szabolcs Kósik

**Affiliations:** 1 Department of Physical Geography, Faculty of Sciences, Eötvös University, Budapest, Hungary; 2 School of Geography, Geology and the Environment, Keele University, Keele, UK; 3 GEOPS, Université Paris-Sud, CNRS, Université Paris-Saclay, Orsay, France; 4 Laboratoire Magmas et Volcans, Université Clermont Auvergne-CNRS-IRD, OPGC, Clermont-Ferrand, France; 5 Faculty of Geology and the Geoenvironment, National and Kapodistrian University of Athens, Athens, Greece; 6 Volcanic Risk Solutions, Institute of Agriculture and Environment, Massey University, Palmerston North, New Zealand

## Abstract

During the Late Bronze Age, the island of Santorini had a semi-closed caldera harbour inherited from the 22 ka Cape Riva Plinian eruption, and a central island referred to as ‘Pre-Kameni’ after the present-day Kameni Islands. Here, the size and age of the intracaldera island prior to the Late Bronze Age (Minoan) eruption are constrained using a photo-statistical method, complemented by granulometry and high-precision K-Ar dating. Furthermore, the topography of Late Bronze Age Santorini is reconstructed by creating a new digital elevation model (DEM). Pre-Kameni and other parts of Santorini were destroyed during the 3.6 ka Minoan eruption, and their fragments were incorporated as lithic clasts in the Minoan pyroclastic deposits. Photo-statistical analysis and granulometry of these lithics, differentiated by lithology, constrain the volume of Pre-Kameni to 2.2–2.5 km^3^. Applying the Cassignol-Gillot K-Ar dating technique to the most characteristic black glassy andesite lithics, we propose that the island started to grow at 20.2 ± 1.0 ka soon after the Cape Riva eruption. This implies a minimum long-term lava extrusion rate of ~0.13–0.14 km^3^/ky during the growth of Pre-Kameni.

## Introduction

**Figure 1:**
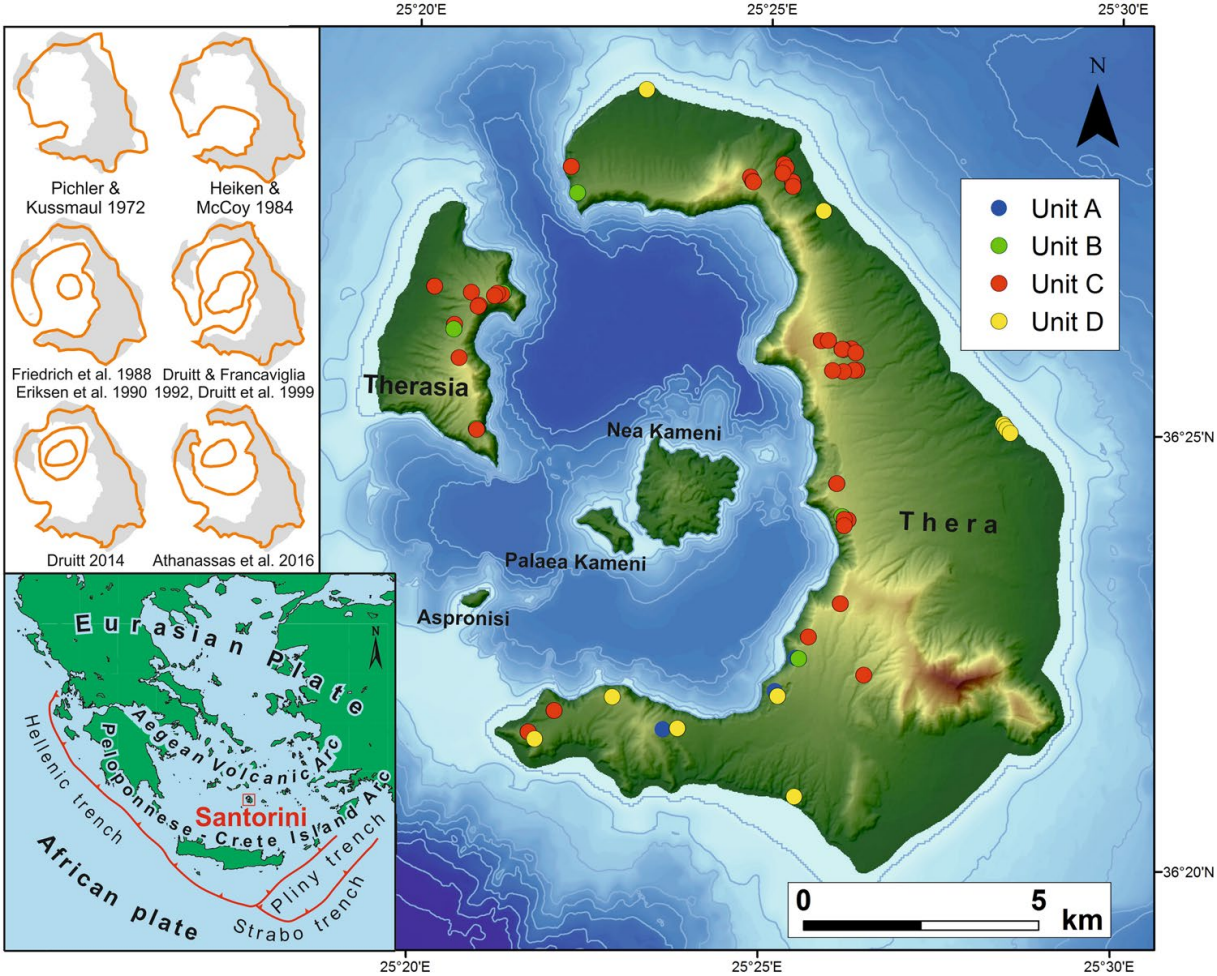
Santorini: tectonic setting and island map with photo-statistical sampling sites. Upper left inset shows the development of Santorini’s pre-Minoan caldera models^6,10,12–17^. Units A to D refer to the deposits of the four phases of the Minoan eruption.

The Late Bronze Age (Minoan) eruption of Santorini (Greece**)**, radiocarbon dated at 1627–1600 BC^1^, was one of the largest eruptions in the Holocene^2^ (Fig. 1). The pyroclastic ejecta deposited on land and on the sea floor around the volcano contain a range of lithic clasts derived from pre-existing volcanic formations. The Minoan eruptive products may comprise up to 117–129 km^3^ bulk volume, corresponding to 78–86 km^3^ DRE^3^ (dense rock equivalent). However, this estimate is partly based on the poorly constrained offshore ignimbrites^4^ and may be regarded as a maximum value.

The landscape of Late Bronze Age Santorini is of great interest to archaeologists, given the presence on the island of the buried Bronze Age town of Akrotiri^5^. It was originally claimed that prior to the eruption there was an island with a central volcanic cone, possibly as high as 500–800 m^6,7^, which was disrupted explosively. The destruction of the central part of the island, proposed already by Fouqué^8^ in 1879, led to caldera formation and resulted in the present-day islands of Thera, Therasia and Aspronisi, which are arranged in a ring around a flooded caldera. Details of caldera formation, and mechanisms of tsunami generation, are discussed by Sparks and Wilson^9^, Druitt^10^, and Nomikou *et al*.^11^.

Heiken and McCoy^12^ contradicted the simple scenario of a collapsed central island and, instead, proposed a caldera model similar to those at Somma-Vesuvius or Krakatau (Fig. 1). However, their proposed caldera in the southern part of the present caldera bay, if it ever existed, was shown by Druitt *et al*.^13^ to have been long filled-in prior to the Late Bronze Age. Friedrich *et al*.^14^ pointed out the occurrence of stromatolites in the Minoan Tuff in the neighbourhood of the northern part of the caldera, interpreting this as evidence of a pre-Minoan shallow bay. Druitt and Francaviglia^15^, Erikssen *et al*.^16^ and recently Athanassas *et al*.^17^ refined the likely extension of the pre-Minoan caldera, which may indeed have been restricted to the northern part of the present-day caldera, possibly with a low, narrow strait to the north^11^ but without an outlet to the southwest.

On the other hand, the presence of abundant lithics, especially clasts of a chemically distinctive black glassy andesite, in the pyroclastic deposits of the Minoan eruption (see below) suggested that the central part of the caldera bay was occupied by an intracaldera island^10,14–16^. We use the term ‘Pre-Kameni’^14,17^ for this pre-Minoan edifice, which may have been similar to the present-day, post-Minoan islands of Palaea and Nea Kameni, but completely destroyed during the Minoan eruption. The size and age of such a Pre-Kameni island have remained uncertain, although broad estimates of 3 to 5 km^3^ were proposed^3,15^ based on the poorly constrained total lithic ejecta^18^. For comparison, the volume of the present-day Kameni Islands is 3.2 km^3^ derived from a merged LiDAR bathymetry grid^19^.

In this paper, by applying a photo-statistical analysis of outcrops complemented by granulometric analysis, we present quantitative results for the volume of the lithic clasts included in the Minoan deposits and, after proportioning their amount, determine the dimensions of the destroyed Pre-Kameni island. The timing of island growth is constrained using high-precision K-Ar dating of the black glassy andesite, which is regarded as the most significant island-forming lithic type^9,10,15^. The topography of Santorini just before the Minoan eruption is also shown by integrating previous and our own results using a high-resolution digitial elevation model (DEM) of Santorini^19^.

### Late-stage evolution of Santorini

The pre-Minoan landscape of Santorini was dominated by a shallow, flooded caldera formed by the last major explosive eruption before the Minoan, the Cape Riva eruption. A synthesis of published dates for the Cape Riva eruption yields a mean age of 21.8 ± 0.4 ka^20^. This eruption is thought to have collapsed the pre-existing Skaros-Therasia lava shield^13,15^. For the subsequent interplinian period which lasted for ~18 ky only minor explosive activity is documented^20–22^.

The 3.6 ka Minoan eruption consisted of four main phases, the deposits of which are denoted A to D by Druitt *et al*.^13^. The first phase was a plinian pumice fall^7,9,23^. A lower, crudely bedded and an upper, unbedded layer, up to 5.5 m thick in total, are collectively named unit A.

During the second phase, interaction with sea water and partial column collapse resulted in phreatomagmatic surges (unit B), ranging in thickness from some dm to up to 12 m^13^.

In the third phase, significant column collapse produced the most prominent unit of the eruption on land (unit C). This is a coarse-grained, massive, up to 55 m thick, phreatomagmatic ignimbrite up to 55 m thick^13^, still reflecting magma-water interaction and deposited at low temperatures^10,24^. The third eruptive phase may have created a tuff cone^3,11^, possibly a large pyroclastic construct (cf.^25^) that filled the caldera bay. This phase is thought to coincide with the explosive disruption of the Pre-Kameni island (along with other parts of Santorini), given the occurrence of abundant, evenly distributed lithic clasts up to 10 m in size in the deposit.

In the fourth phase, finer grained, hot ignimbrite (unit D) was produced from multiple vents without phreatomagmatic character. This phase may have been coeval with major caldera collapse^9,10^. The unit thickens distally up to 80 m offshore and is thought to be the most voluminous Minoan deposit^4^. The ignimbrites contain thin lithic concentration zones and are capped (or cut) in some places by lithic-rich gravelly beds of fluvial origin up to 5–10 m thick^10,13^. The presence of the latter indicates considerable potential energy as well as transport distance required for fluvial redeposition of the lithics contained in unit C, B and A, implying a topography which may have been truncated by the caldera collapse only toward the end of phase 4.

## Materials, Methods and Results

### Petrography and geochemistry of lithic clasts in the Minoan deposits

**Figure 2:**
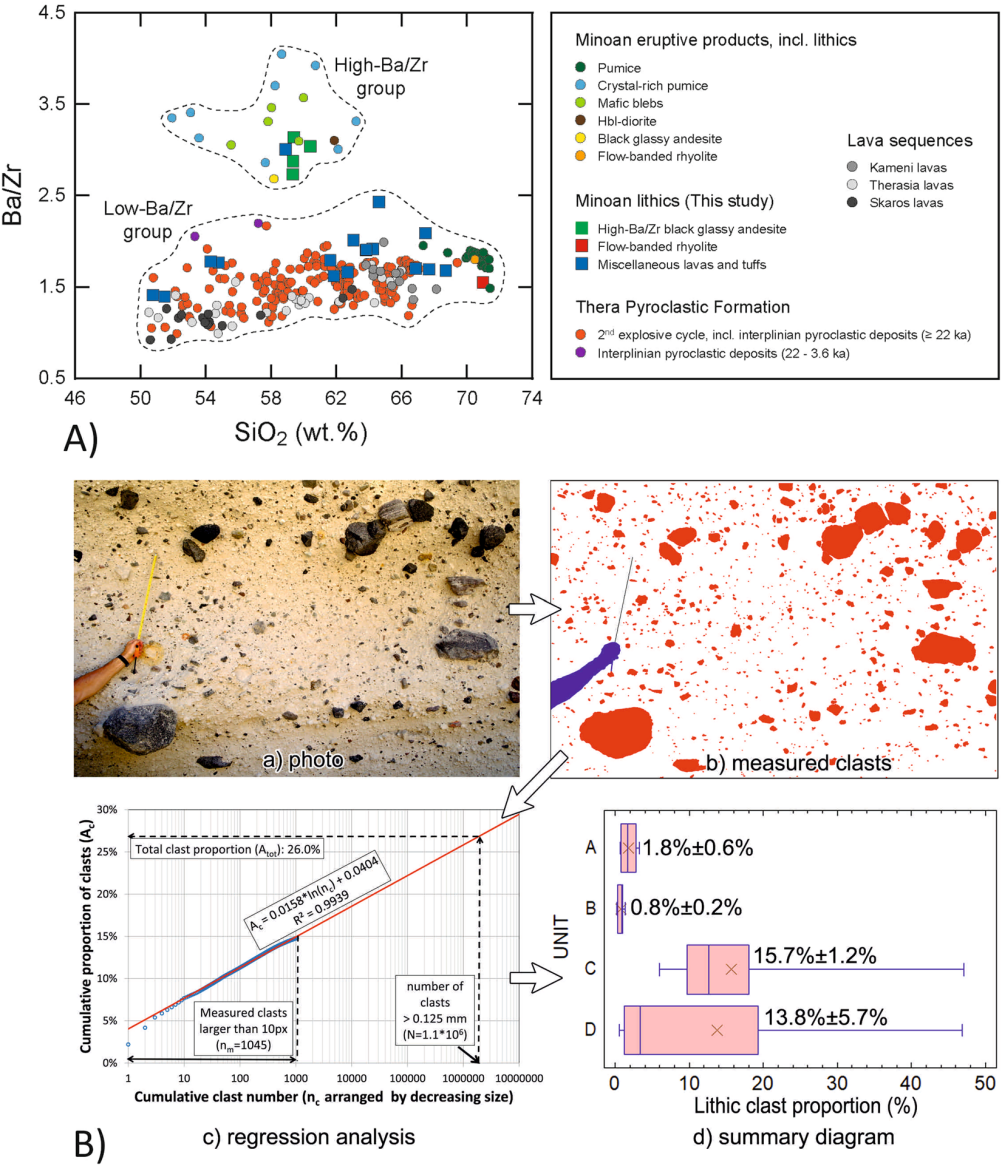
(**A**) Ba/Zr vs SiO_2_ (wt.%) plot of lithic clasts in the Minoan deposits (this study) compared with published data for the Minoan eruptive products (including lithic clasts), the Thera Pyroclastic Formation (2^nd^ explosive cycle), and Santorini lava sequences. Data sources: Druitt^10^, Druitt *et al*.^13^, Fabbro *et al*.^20^, Vespa *et al*.^21^, Vaggelli *et al*.^22^, Barton *et al*.^28^, and Huijsmans and Barton^29^. (**B**) Photo-statistical analysis of lithic content: (a, b) methodology, (c) cumulative clast area vs cumulative clast number on a sample diagram, (d) summary diagram of lithic proportions in units (**A**–**D**) plotted in a box-whisker diagram (numbers are mean ± standard error).

The Minoan deposits contain a diverse range of juvenile components as well as lithic clasts derived from older parts of Santorini^10,13^. On the basis of Druitt^10^, the lithic clasts analysed here are grouped into black glassy andesite, flow-banded rhyolite, and miscellaneous lavas and tuffs (Fig. 2A). Geochemically, the Minoan lithics range from basalt to rhyolite (50.8–71.0 wt.% SiO_2_). In addition, a dominant low-Ba/Zr group of lithics is distinguished from a characteristic high-Ba/Zr group of clasts of mainly intermediate (andesitic) composition, which are unique in the eruptive history of Santorini since 530 ka^10^. Among the high-Ba/Zr group, a conspicuous suite of fresh, black, glassy and porphyritic blocks of andesite lava (BGA hereafter) has been interpreted as fragments derived from a pre-Kameni edifice^7,9,10^. This high-Ba/Zr BGA is distinguished from similar blocks of dark-grey, porphyritic andesite of low-Ba/Zr nature interpreted as fragments of older lava sequences, such as the Therasia lavas^13,20^. Blocks characterised by a high Ba/Zr ratio also include a variety of slightly altered, light-grey and fine-grained andesite lava and an altered clast of dacitic tuff, grouped under ‘Miscellaneous lavas and tuffs’ (Fig. 2A). A likely origin of these clasts is the early centres of the Akrotiri peninsula (650–550 ka^10,13^). From the above, it can be concluded that the high-Ba/Zr BGA is the dominant lithology that constituted the Pre-Kameni island.

### Volumetric analysis of the lithic clasts in the Minoan deposits

76 sites (Fig. 1) have been selected for photo-statistics of the lithic clasts in order to infer the volume of Pre-Kameni. Unit C, with dispersed lithic clasts, is represented by 58 sites; the lithic-poor units of A and B by 4 and 5 sites, respectively, and unit D by 9 sites. The latter includes both the ash-rich, lithic-poor facies and the thin lithic concentration horizons in the ignimbrites. Details of the photo-statistical method are given in Fig. 2B and Appendix 1, and the numerical results are summarized in Table 1.

**Table 1:** Result of the volumetric analysis of the Pre-Kameni island and total lithic content (vol%) in the Minoan deposits, using photo-statistical data, volume data from Bond and Sparks^7^, Johnston *et al*.^3^, Pyle^18^, Sigurdsson *et al*.^4^ and Watkins^30^, and black glassy andesite proportions from Druitt^10^.

Unit	Volume (km^3^)		Lithic clast vol% from photo-statistics	of which black glassy andesite %	Volume of total lithic content (km^3^)		Volume of ‘Pre-Kameni’ (km^3^)	
*A*	37.6		1.8 ± 0.6	3.8 ± 0.9	0.67 ± 0.28		0.03 ± 0.01	
*B*	7.8		0.8 ± 0.2	16.8 ± 2.8	0.06 ± 0.02		0.01 ± 0.00	
*C*	25.9	15.5	15.7 ± 1.2	30.4 ± 3.1	4.06 ± 0.52	2.43 ± 0.31	1.23 ± 0.16	0.74 ± 0.09
*D*	51.7	62.1	13.8 ± 5.7	10.8 ± 1.0	7.14 ± 3.00	8.57 ± 3.61	0.77 ± 0.33	0.93 ± 0.39
*sum*	123				11.93 ± 3.82	11.74 ± 4.22	2.04 ± 0.50	1.70 ± 0.50
*additional lithics* ^1^	123*40% = 49.2		10	10	4.92		0.49	
*total*	123				16.85 ± 3.82	16.66 ± 4.2	2.53 ± 0.50	2.19 ± 0.50

Left panels in divided cells show values assuming that the ratio of C:D is 1:2; right panel assuming that the ratio of C:D is 1:4. For the given error estimates see text. For methodology^31–39^, see Appendix 1 in supplementary material.

^1^Additional lithics from the finest (<0.125 mm) fraction of all units resulting from sieving.

Although the clast proportion values of the 58 sites of unit C show a great dispersion, the average percentage of the lithic clasts (15.7 vol%) is statistically confident as the standard error for unit C is quite small (1.2%). As for the other units, they are undersampled, but the studied locations are considered to be representative. In units A and B, the percentage of lithic clasts is insignificant (1.8 and 0.8 vol%, respectively), so the standard error is also small (0.6% and 0.2%). In contrast, the average clast proportion increases to 13.8 vol% in unit D (with 5.7% standard error), and the data are even more scattered than in case of unit C. This fact is in accordance with the occasional occurrence of lithic-rich beds. All clast proportions were calculated for the >0.125 mm fraction (see Fig. 2B and Appendix 1).

Granulometric analysis of 4 samples, one from each unit, shows that the finest fraction of <0.125 mm diameter represents an important addition: 39–41 wt% of the bulk material (except the sample from unit A where it is only 6.5%). The proportion of BGA was also measured manually in each fraction of the sieved samples, and in the coarse fraction (>16 mm) of three other unit C locations. Results show that, for unit C, the proportion of BGA ranges between 18% and 31% in the coarsest fraction, whereas in the finer fractions (16–0.125 mm) it varies between 15.9 and 45.1%. These values support that the mean BGA proportion of unit C can be appropriately taken as 30% as proposed by Druitt (2014). As for the other units, we obtained 4.0–18.2% for the BGA proportion in unit D and 0–26.3% in unit A taken all fractions into account, and we did not find BGA clasts in the studied sample of unit B.

In general, both the proportion of lithics and the proportion of BGA within the lithics show a decreasing trend with clast diameter. Based on this trend, we propose that both the proportion of lithics and the proportion of BGA within the lithics in the finest (<0.125 mm) fraction is less than 10%. Accordingly, in our study we calculated with 10% as a maximum value.

### K-Ar dating of the black glassy andesite lithic clasts

**Figure 3:**
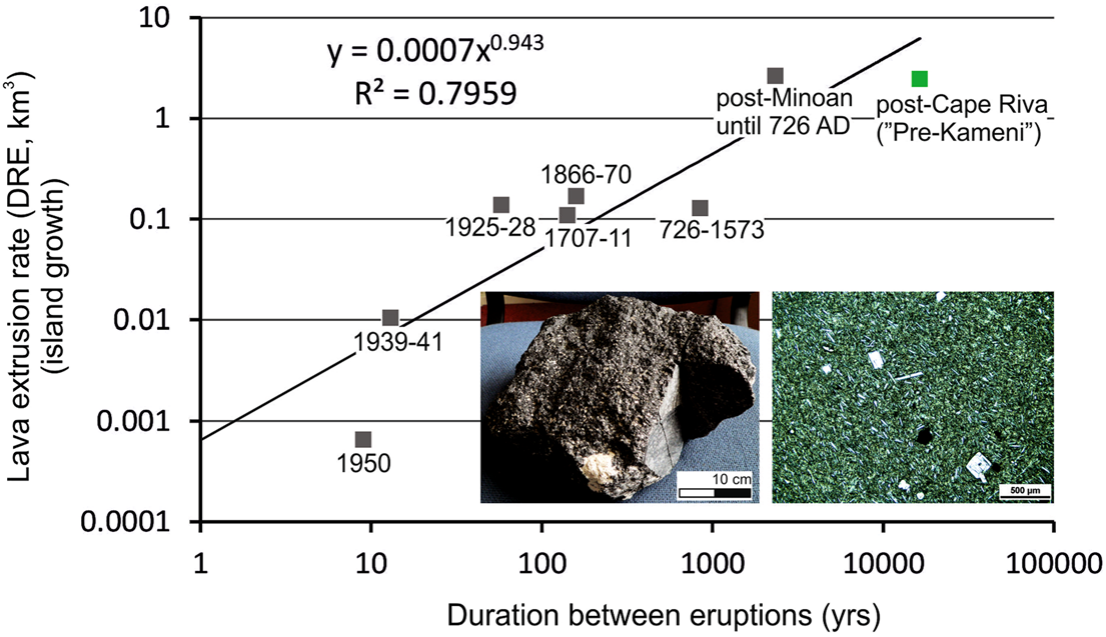
Lava extrusion vs time at Santorini’s central lava dome islands in post-Cape Riva times (post-Minoan volumes are from Nomikou *et al*.^19^. Green quadrangle marks the proposed Pre-Kameni, gray quadrangles the Post-Minoan eruptions. Log scale has been used because the inter-eruption durations and lava extrusion values have several order of magnitude differences. Since the exponent in the equation is close to 1, the relationship between lava extrusion and elapsed time is quasi-linear. Insets: sample photograph (right) and thin section image (left, in PPL) of the main constituent of the Pre-Kameni island, the black glassy andesite (BGA).

K-Ar dating of a single BGA sample was performed in the GEOPS laboratory (Orsay, France) using the unspiked Cassignol-Gillot technique, which is especially suitable for dating young samples with low radiogenic argon content (Gillot *et al*.^26^; for details, see Appendix 2). The ^40^Ar-^39^Ar dating technique was not preferred for the sample, because the irradiation may induce significant ^39^Ar recoil from the vitrous groundmass and a high production of interferring ^36^Ar from Ca. Five independent argon analyses of different aliquots of the sample yielded ages ranging between 18.7 ± 3.1 and 21.5 ± 2.0 ka leading to a weighted mean (using the inverse of the variance)^27^ of 20.2 ± 1.0 ka (Table 2, Fig. 3).

**Table 2:** K-Ar ages performed on groundmass separate of sample 15SAN-A (a 3-kg black glassy andesite lithic block from unit C of the Minoan Tuff).

**Sample**	^40^Ar* (%)	^40^Ar* (×10^10^ at./g)	Age ± 1σ (ka)
15SAN-A	0.6	4.1278	18.7 ± 3.1
(K%: 2.11 ± 0.02)	1.0	4.4882	20.4 ± 2.0
	1.1	4.7406	21.5 ± 2.0
	1.0	4.4235	20.1 ± 2.0
	1.0	4.2728	19.4 ± 2.0
		Weighted mean:	20.2 ± 1.0

Column headings indicate concentration of radiogenic ^40^Ar (^40^Ar*) in percent; concentration of radiogenic ^40^Ar (^40^Ar*) × 10^10^ in number of atoms per gram; age ± 1-sigma uncertainty of each measurement (in ka), and weighted mean age and uncertainty (see text)^40–45^.

## Discussion

While the caldera morphology of Santorini prior to the Minoan eruption has been recently clarified^17^ building on the earlier data of Druitt and Francaviglia^15^, the size and age of a Pre-Kameni island have remained tentative. The rationale for our analysis is that a significant fraction of the lithic clasts, predominantly the BGA included in the Minoan deposits, represents Pre-Kameni, whereas the rest is derived from other destroyed parts of Thera and Therasia. Moreover, dating the BGA can give insights into the timing of Pre-Kameni relative to the Cape Riva eruption.

In order to estimate the volume of the pre-Minoan intra-caldera volcanic island, we proportioned the lithic content between the four Minoan units. At first we considered the maximum estimate of 60 km^3^ DRE of the on-land and offshore pyroclastic material^4^. Of this, 41 km^3^ DRE submarine pyroclastic flow deposits (mostly unit D) corresponds to 54.5 km^3^ bulk volume^4^, i.e. the ratio of volume to DRE is 1.33:1. Using this ratio, the on-land DRE values^4^ correspond to the following bulk volumes: Plinian fall: 3 km^3^ (2 km^3^ DRE), on-land ignimbrite: 2 km^3^ (1.5 km^3^ DRE), and offshore co-ignimbrite ash: 22.5 km^3^ (17 km^3^ DRE), totalling 82 km^3^ bulk volume.

Additional 18–26 km^3^ DRE (31–41 km^3^ bulk volume, i.e. 36 km^3^ on average) of the intra-caldera infill was proposed by Johnston *et al*.^3^, representing units A, B and C (unit D is considered negligible within the caldera). As for their relative proportions, we used the isopach values published by Bond and Sparks^7^, and Heiken and McCoy^12^. Units A, B and C have average thicknesses of 2 m, 5 m, and 25 m, respectively, i.e. they represent 6%, 16%, and 78% of the intra-caldera infill. Dividing the bulk volume estimate of Johnston *et al*.^3^ by these percentages gives 2.0–2.6 km^3^ for unit A, 5.0–6.6 km^3^ for unit B, and 25.0–32.8 km^3^ for unit C, which are included in the volume values of the Minoan deposits given in Table 1.

This proportioning, along with the ~40% extra lithic content of the finest fraction from granulometric analysis, makes it possible to calculate the total lithics contained in each unit (see Table 1), as well as the BGA (i.e. Pre-Kameni island) volume within the units. An unsolved question is how to divide the 54.5 km^3^ bulk volume of submarine pyroclastic flow deposits^4^ between unit C and D. As unit C decreases rapidly in thickness away from the caldera, we propose a ratio of C:D = 1:2 or even 1:4 (both calculations are given). The final standard errors in Table 1 include uncertainty resulting from lithic clast proportions and BGA proportions, but do not take into account the uncertainty of deposit volumes and additional lithics proportion, because the latter values are estimates from the published literature.

As our results show, the total volume of the lithics in the Minoan deposits is ~16–17 km^3^, more than twice the value suggested by Pyle^18^. However, within this, the volume of Pre-Kameni, if we envisage that the island was made up entirely of BGA, is significantly smaller, ranging between 2.2 km^3^ (C:D = 1:4) and 2.5 km^3^ (C:D = 1:2). As for the calculated range, we consider that the smaller value of 2.2 km^3^ (i.e. a lower C:D ratio) is more realistic due to the overwhelming presence of unit D in submarine settings. However, assuming that parts of Pre-Kameni may have collapsed into the caldera and were not incorporated into the Minoan deposits, this smaller value must be considered a minimum estimate. This may be offset against a possible, albeit unconstrained, decrease in lithic concentrations with distance from source and uncertainties about the precise volume relationships between units C and D. However, it is the unit C ignimbrite which contains the evenly distributed BGA clasts. Unit D, which is thought to be coeval with caldera collapse during the Minoan eruption, contains lithic clasts of BGA interpreted as predominantly remobilised from unit C. Therefore, it can be inferred that most of the Pre-Kameni island, which was located close to the main vents, was incorporated in unit C.

Such a Pre-Kameni island, on the basis of a new K-Ar age obtained by the K-Ar Cassignol-Gillot technique, seems to have started to grow immediately after the Cape Riva eruption at 20.2 ± 1.0 ka, similar to the present-day Kameni Islands after the Minoan catastrophe^11,13^. However, the obtained age does not constrain the duration of island growth. The extrusive activity may have declined, and possibly the island was dormant by the Late Bronze Age. This would be consistent with the lack of clasts of BGA with radial jointing (indicative of hot emplacement) in the Minoan products^10^. Nevertheless, this age allows us to calculate a minimum long-term mean lava extrusion rate for the period from 20.2 to 3.6 ka assuming a constant activity: considering the 2.2–2.5 km^3^ island volume, the rate is 0.13–0.14 km^3^/ky. However, because this long-term value is only one seventh of the average growth rate during the much shorter lifetime of the present-day Kameni Islands (~0.9 km^3^/ky since the Minoan eruption^19,28^; Fig. 3), it is also possible that Pre-Kameni grew up in a short period at higher lava extrusion rates.

**Figure 4:**
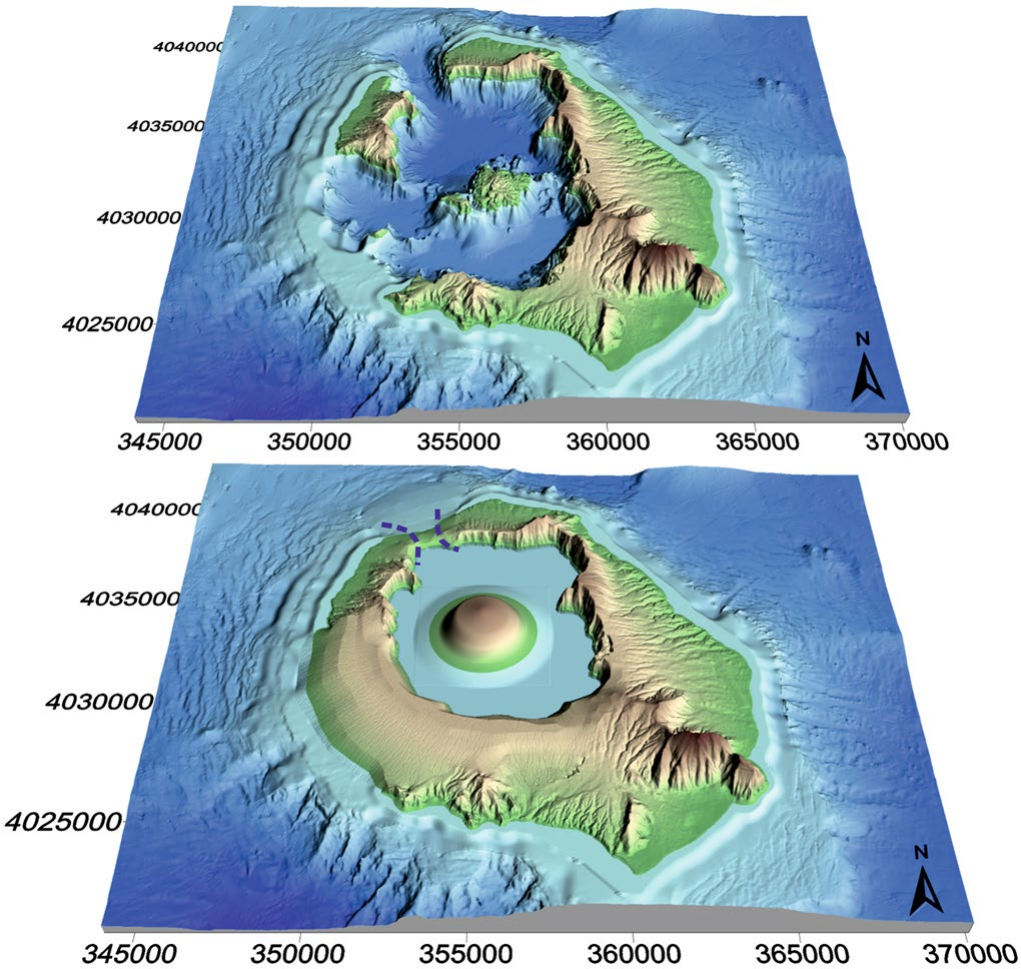
Digital elevation model (DEM) reconstruction of Santorini comparing (**a**) the present-day topography and (**b**) the proposed topography prior to the Minoan eruption. The latter shows the reduced size of a Pre-Kameni island, a smaller and shallower caldera harbour restricted to the north, a possibly smaller caldera outlet (dotted blue line) in the north, and a continuous southern caldera rim connecting Thera and Therasia through Aspronisi.

Finally, we visualize the proposed physiography Santorini prior to the Minoan eruption (Fig. 4) using a combined DEM with 10 m resolution offshore and 15 m resolution onshore^11^. Here, an unconstrained strait in the north, a closed caldera rim in the southwest, a smaller caldera bay (after filling a large part of the present-day southern caldera basin Druitt^10^; Nomikou *et al*.^11^; Athanassas *et al*.^17^), and a 2.4 km^3^ Pre-Kameni island (with ideal low dome shape) in the centre of the flooded bay, are depicted. The SW caldera rim was completed by adding some contours with interpolation, in order to connect Thera and Therasia. The presented DEM reconstruction, i.e. the proposed 3D view of Minoan Santorini, is considered a combination of the obtained quantitative results and qualitative information on the Late Bronze Age topography.

## Electronic supplementary material

Supplementary material
