# Supplementary material for: Towards reconstruction of the lost Late Bronze Age intra-caldera island of Santorini, Greece
Source: Sci Rep. 2018 May 4;8:7026. doi: 10.1038/s41598-018-25301-2 (PMC5935677; doi:10.1038/s41598-018-25301-2)
Supplement: Supplementary file 1 — Supplementary material [file 41598_2018_25301_MOESM1_ESM.doc]

**Supplementary material**

**Toward the reconstruction of the disappeared Late Bronze Age intra-caldera island of Santorini, Greece**

Dávid Karátson, Ralf Gertisser, Tamás Telbisz, Viktor Vereb, Xavier Quidelleur, Timothy Druitt, Paraskevi Nomikou, Szabolcs Kósik

***APPENDIX 1. The photo-statistical method***

Textural characteristics and clast volumetric proportions of sedimentary and pyroclastic deposits can be quantified by photo-statistical methods (e.g. Capaccioni and Sarocchi^31^; Karátson et al.^32^; Armienti^33^; Shea et al.^34^; Sarocchi et al.^35^; Jutzeler et al.^36^). In this study, photos were taken from 5-10 m distance **(Fig. 2).** A geometrical adjustment and scaling were applied to each photo, then the lithic clasts in the photos were outlined using a semi-automated method based on intensity differences between the clasts and the matrix (Jutzeler et al.^36^). At first, clasts larger than 10 pixels (~0.1 cm^2^ in real size depending on photo distance) were outlined, and their number (*n_m_*: number of measured clasts) and areas were measured. We plotted the cumulative area (*A_c_*) of measured clasts (as a proportion of the total image area) against the cumulative clast number (*n_c_*). We found a very strong logarithmic correlation between cumulative clast area and cumulative clast number (the minimum *r^2^* is *0.912* in our samples), which is attributed to the fragmentation of clasts during explosion resulting in a Weibull distribution (e.g. Wohletz and Brown^37^). Given the logarithmic nature of this relationship, it implies that although the smaller grains are more numerous (by several order), their areal and hence volumetric proportion is relatively small, but not negligible (see **Fig. 2**), which must be taken into account e.g. by granulometry**.** Another strong power law relationship exists between the individual area of clast ordered decreasingly and the cumulative clast number. This second function was used to calculate the number (*N*) of clasts (with diameter >0.125 mm). Based on the above functional relationships and the estimated clast number, we calculated the total clast area proportion (*A_tot_*) using the following equation:

*A_tot_ = a⋅ln(N) + b ,*

where *a* and *b* are equation parameters distinctive for each photo. Given the Delesse principle (Baddeley and Jensen^38^), the total clast area proportion equals the volumetric proportion of lithic clasts within the deposit.

***APPENDIX 2. The Cassignol-Gillot K-Ar technique***

For dating, a large fresh block of the glassy andesite was sampled, and following a careful mineralogical separation a pure groundmass fraction (of 125-250 µm grains) was selected. Heavy liquids were used to keep the groundmass in a narrow density range of 2.52-2.58 g/cm^3^ in order to eliminate possible undetected traces of weathering. Finally, a magnetic separator was used to further improve purity of the fraction by removing possible residual phenocrysts. Potassium (K) content was determined by flame spectroscopy and compared to reference standards MDO-G (K=3.51%; Gillot et al.^29^) and BCR-2 (K= 1.486%; USGS Standard). The relative uncertainty of the K measurement ($\sigma_{K}$) is ~ 1%. Argon (Ar) was measured using a multi-collector 180° sector mass spectrometer similar to the one described in Gillot and Cornette^40^. For young samples, such as the one dated here, the atmospheric correction introduces a relatively large uncertainty ($\sigma_{^{40}Ar*}$). It is calculated using the following formula:

$\sigma_{^{40}Ar*}=\frac{0.1}{\%^{40}Ar*}$,

as the detection limit of the radiogenic argon content ($\%^{40}Ar*$) in our system is 0.1% (Quidelleur et al.^41^).

Details of the whole procedure are given in Germa et al.^42^. When including all sources of uncertainties, the relative uncertainty for each age determination was calculated using

$\sigma_{t}=\sqrt{\sigma_{K}^{2}+\sigma_{^{40}Ar_{cal}}^{2}+\sigma_{^{40}Ar*}^{2}}$ , with $\sigma_{^{40}Ar_{cal}}$ ,

the relative uncertainty associated with the argon calibration obtained from routine analyses of the HD-B1 standard (Hess and Lippolt^43^).

Since only two argon isotopes (^40^Ar and ^36^Ar) are measured for (1) the sample, (2) the atmospheric correction and (3) the signal calibration, no mass discrimination correction is needed with the Cassignol-Gillot technique. Furthermore, the knowledge of the exact ^40^Ar/^36^Ar atmospheric ratio here is unnecessary. K isotopic ratios and ^40^K decay constants of Steiger and Jäger^44^ have been used.
